# Online immunocapture ICP-MS for the determination of the metalloprotein ceruloplasmin in human serum

**DOI:** 10.1186/s13104-018-3324-7

**Published:** 2018-04-02

**Authors:** Bogdan Bernevic, Ahmed H. El-Khatib, Norbert Jakubowski, Michael G. Weller

**Affiliations:** 1 Division 1.5 Protein Analysis, Federal Institute for Materials Research and Testing (BAM), Richard-Willstätter-Strasse 11, 12489 Berlin, Germany; 2 Division 1.1 Inorganic Trace Analysis, Federal Institute for Materials Research and Testing (BAM), Richard-Willstätter-Strasse 11, 12489 Berlin, Germany; 3 Faculty of Pharmacy, Department of Analytical Chemistry, Ain Shams University, Organization of African Unity Street, Abassia, Cairo, 11566 Egypt

**Keywords:** Ceruloplasmin, Immunocapture, ICP-MS, ELISA, Human serum

## Abstract

**Objective:**

The human copper-protein ceruloplasmin (Cp) is the major copper-containing protein in the human body. The accurate determination of Cp is mandatory for the reliable diagnosis of several diseases. However, the analysis of Cp has proven to be difficult. The aim of our work was a *proof of concept* for the determination of a metalloprotein-based on online immunocapture ICP-MS. The immuno-affinity step is responsible for the enrichment and isolation of the analyte from serum, whereas the compound-independent quantitation with ICP-MS delivers the sensitivity, precision, and large dynamic range. Off-line ELISA (enzyme-linked immunosorbent assay) was used in parallel to confirm the elution profile of the analyte with a structure-selective method. The total protein elution was observed with the ^32^S mass trace. The ICP-MS signals were normalized on a ^59^Co signal.

**Results:**

The human copper-protein Cp could be selectively determined. This was shown with pure Cp and with a sample of human serum. The good correlation with off-line ELISA shows that Cp could be captured and eluted selectively from the anti-Cp affinity column and subsequently determined by the copper signal of ICP-MS.

**Electronic supplementary material:**

The online version of this article (10.1186/s13104-018-3324-7) contains supplementary material, which is available to authorized users.

## Introduction

Ceruloplasmin (Cp) is an enzyme belonging to the multi-copper oxidase family and contains six copper atoms [1, 2]. In human plasma from healthy subjects, more than 95% of the total copper is bound to Cp. Serum Cp levels of less than 200 mg/L are considered to be a diagnostic criterion for Wilson’s disease [3], an autosomal recessive inherited disorder of copper metabolism, which can be fatal, if not treated properly. In addition, the Menkes disease [4, 5] (“kinky hair syndrome”) can be confirmed by determination of Cp.

The diagnostic determination of Cp in serum or plasma is usually performed by turbidimetric or nephelometric [6] and other immunoassays [7]. In addition, some other methods have been published, such as SEC–ICP-MS [8], where a depletion cartridge was used to remove highly abundant proteins, such as albumin. In any case, standardization of Cp analysis turned out to be quite difficult and left some serious questions unanswered largely due to the lack of Cp reference materials. To resolve these issues, we explored the feasibility of an approach based on an immunocapture step, which was performed as clean-up and enrichment, followed by hetero-element detection by use of inductively coupled plasma mass spectrometry (ICP-MS).

Immunocapture, which is a variant of affinity extraction or affinity chromatography, can be regarded as one of the most powerful separation techniques available [9–11]. This approach is particularly valuable when the analyte is present in low concentrations, and the matrix is complex, such as in food analysis or human diagnostics. In the field of high-sensitivity protein analysis, usually only affinity-based techniques are feasible, e.g. enzyme-linked immunosorbent assay (ELISA) [12]. Unfortunately, the calibration of immunoassays is not trivial and many inexperienced users have problems to interpret the results properly. Particularly, the existence of “cross-reactivity” often leads to some confusion, although this is nothing else as an analytical interference, which is occurring in any instrumental method. Affinity extraction is often combined with instrumental analytical techniques, such as mass spectrometry [13, 14] and hence does not need much rethinking in relation to a more traditional analytical workflow. However, the most selective affinity techniques are commonly based on antibody/analyte interactions, requiring sufficient amounts of high-quality antibodies directed against the respective analyte. The availability of such antibodies may be limited and almost always the cost of a sufficient amount of antibodies is high. On the other hand, in the field of protein analysis, even researchers with many years of experience sometimes seem to underestimate the complexity of their samples and therefore do not sufficiently appreciate the importance of extensive sample-preparation steps.

In this work, we used an uncommon approach to produce sufficient amounts of antibodies in chicken eggs at a reasonable cost in an animal-friendly way. The developed antibodies were used for the enrichment and isolation of Cp, followed by its quantitative determination using ICP-MS.

## Main text

### Experimental setup

**Fig. 1:**
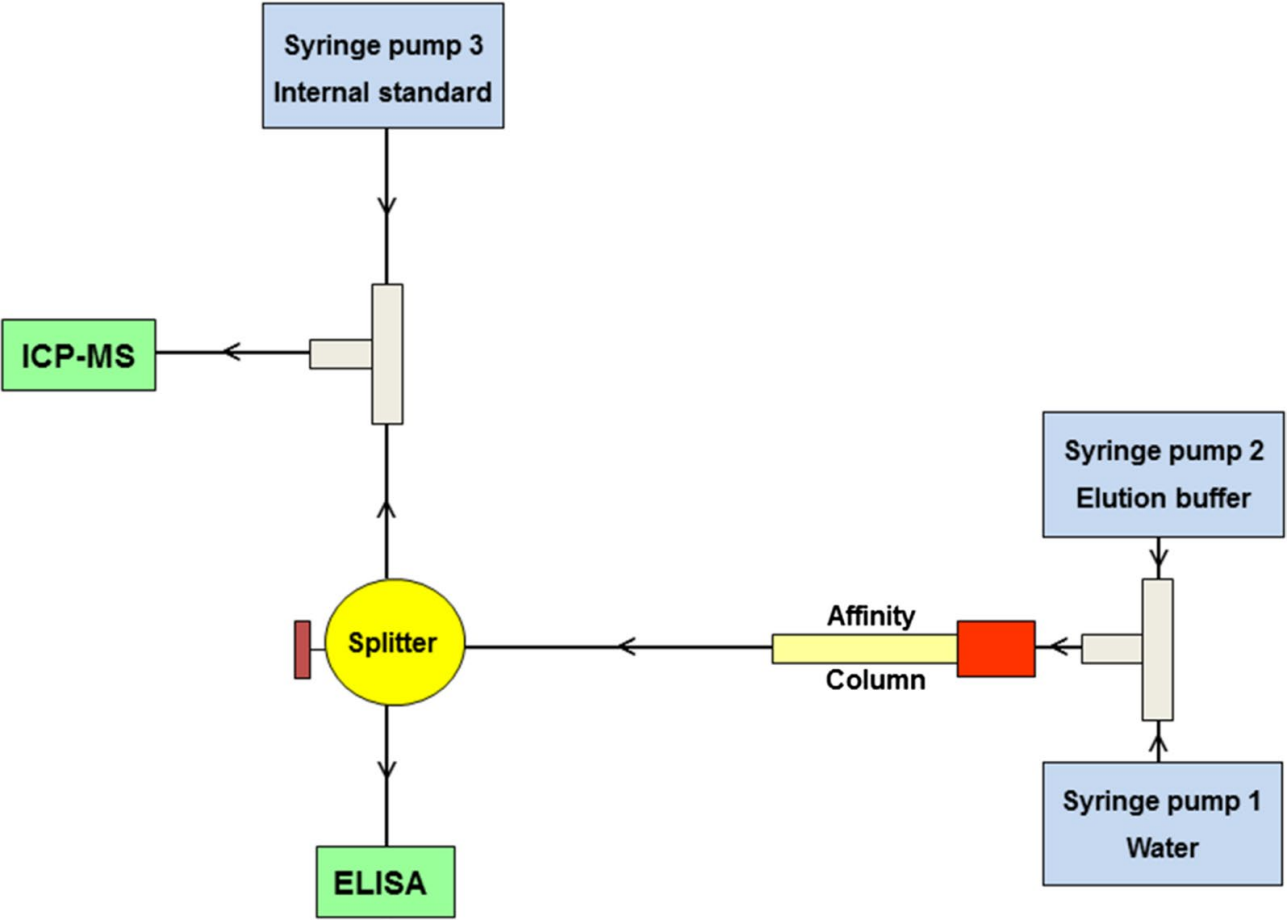
Schematic representation of the immunocapture ICP-MS setup

The schematic representation of immunocapture ICP-MS setup is shown in Fig. 1. The eluate was split into two equal parts for the collection on an ELISA plate as well as online ICP-MS analysis. More experimental details can be found in Additional files 1 and 2.

## Results

**Fig. 2:**
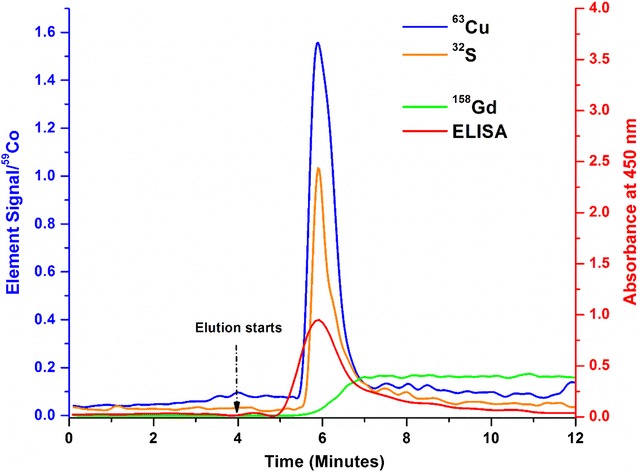
Immunocapture elution signals of an injection of pure, human ceruloplasmin (90 µg). All ICP-MS signals were normalized to ^59^Co. ^158^Gd was a tracer for the elution buffer

In Fig. 2, ICP-MS data show elution peaks of the characteristic elements expected for pure Cp (S, Cu). The signals were normalized to ^59^Co, which was introduced continuously as a post-column internal standard. The stability of the elution process was demonstrated by the tracer (^158^Gd) which is not retained on the column and was therefore introduced in the elution buffer. Generally, ELISA and ICP-MS data showed comparable elution profiles.

A tentative quantification of Cp was attempted through comparing the ICP-MS signal of copper in Cp with that of standard elemental copper. The recovery of Cp was 94% (based on the nominal Cp concentration and 6 copper atoms per Cp).

**Fig. 3:**
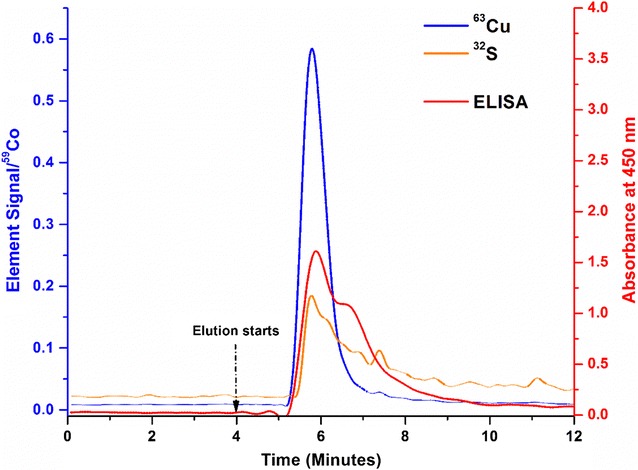
Immunocapture elution signals of an injection of human serum containing a natural level of ceruloplasmin (100 µL diluted with 10 mL of PBS). The ICP-MS signals were normalized to ^59^Co

In Fig. 3, the injection and elution of a real sample (human serum) are shown. The sulfur and the Cp-selective ELISA traces are somehow broadened, which is an indication that the elution of the protein part of the molecule is delayed by slower diffusion or non-specific binding. Due to the fact that the Cu signal is not affected, the shoulder in the sulfur signal is a hint for eluting serum proteins or of Cp fragments, which do not contain any copper. Considering that the quantitation of Cp should be achieved finally, the result is very interesting. Although the protein and the metal peaks are separated slightly, accurate quantitation based on ICP-MS should be achieved without difficulty. By this experiment, it can be demonstrated that the immunoaffinity approach is robust and can be applied to complex serum samples as well. Comparison with the ELISA results also demonstrates that Cp is eluted more or less as an intact protein. Using 100 µL of serum, the amount of Cp in the serum is calculated to be 166 µg (using standard copper solution and based on 6 copper atoms in Cp). Validation of the new method can be achieved once a certified reference material (CRM) of Cp will become available.

## Discussion

The selective detection of the copper-protein ceruloplasmin was achieved by an immunoaffinity ICP-MS approach. It could be shown that ceruloplasmin can be eluted by a glycine/HCl buffer (pH 2.2) and subsequently detected and tentatively quantified online in an ICP-MS system. For validation purposes, a fractionated immunoassay was performed. The elution curves monitored by ICP-MS and immunoassay correlate to a high extent and prove that copper and the protein part of the ceruloplasmin can be eluted from the affinity column. However, for the quantification of ceruloplasmin by ICP-MS it is irrelevant, whether copper is still bound to the ceruloplasmin in the elution buffer. The peak area of copper represents the amount of ceruloplasmin considering its stoichiometry. The copper:sulfur ratio is a good way to identify copper losses because sulfur is covalently bound to the protein, whereas copper may be exchanged or lost to a certain extent. Critical for the success of this approach is the use of buffers of extremely low metal content. Nevertheless, the introduction of an additional washing step with ultrapure lab water reduces the background signal by a factor of 10 and also facilitates the integration of the peaks.

## Limitations

This work should be seen as a *proof of concept*. Validation and exploration of the applicability to other metalloproteins or sample types have to be performed in the future. Nevertheless, this work shows that online immunocapture ICP-MS is an interesting option for quantitative protein analysis.

## Additional files

**Additional file 1.** Table of the instrumental parameters of ICP-MS analysis.

**Additional file 2.** Experimental details. Preparation of chicken antibodies against human ceruloplasmin. ELISA protocol for antibody testing and offline ceruloplasmin determination. Preparation of the immunocapture affinity column. ICP-MS protocol. Protocol of the immunocapture ICP-MS experiments.

## Abbreviations

Cp: ceruloplasmin
CRM: certified reference material
ELISA: enzyme-linked immunosorbent assay
HCl: hydrochloric acid
ICP-MS: inductively coupled plasma mass spectrometry
SEC: size exclusion chromatography
