# Supplementary material for: Online immunocapture ICP-MS for the determination of the metalloprotein ceruloplasmin in human serum
Source: BMC Res Notes. 2018 Apr 2;11:213. doi: 10.1186/s13104-018-3324-7 (PMC5879926; doi:10.1186/s13104-018-3324-7)
Supplement: Supplementary file 1 — Additional file 1. Table of the instrumental parameters of ICP-MS analysis. [file 13104_2018_3324_MOESM1_ESM.pdf]

Additional File 1:

Instrumental parameters of ICP-MS analysis

Table S1

RF plasma power	1250 W
Plasma gas flow (Ar)	16 L min^{-1}
Auxiliary gas flow (Ar)	1.1 L min^{-1}
Sample gas flow (Ar)	0.72 L min^{-1}
Mass resolution ($m/\Delta m$)	4000 (MR)
Sample time	10 ms
Scanning mode	E-scan
Detected isotopes	^{32}S, ^{59}Co, ^{63}Cu, ^{65}Cu, ^{168}Gd
Tracer in elution buffer	^{158}Gd
Post-column internal standard	^{59}Co
Nebulizer	Glass concentric nebulizer (Thermo)
Spray chamber	Quartz cyclonic spray chamber Twinnabar (Thermo)
