# Supplementary material for: Online immunocapture ICP-MS for the determination of the metalloprotein ceruloplasmin in human serum
Source: BMC Res Notes. 2018 Apr 2;11:213. doi: 10.1186/s13104-018-3324-7 (PMC5879926; doi:10.1186/s13104-018-3324-7)
Supplement: Supplementary file 2 — Additional file 2. Experimental details. Preparation of chicken antibodies against human ceruloplasmin. ELISA protocol for antibody testing and offline ceruloplasmin determination. Preparation of the immunocapture affinity column. ICP-MS protocol. Protocol of the immunocapture ICP-MS experiments. [file 13104_2018_3324_MOESM2_ESM.pdf]

# Additional File 2:

# Experimental Details

## Materials and Methods

The polyclonal antibodies were produced by immunization of chicken, which has the advantage that IgY (avian) antibodies can be collected from the egg yolk and not from the animal's blood. In addition, most human proteins, such as ceruloplasmin, show a high immunogenicity in chicken. The egg yolk was purified in a two-step approach, which was performed by the company in charge. The immunization was performed by repeated injection of commercial Cp according to the company's standard protocol. The crude IgY was further purified on an affinity column, made by immobilization of the commercial Cp on an activated column. The pre-purified raw extract from egg yolk was applied on this column and the IgY was eluted by an elution buffer based on citrate/glycine (pH 2.8). The eluent was neutralized immediately with phosphate buffer (pH 8) and - after a buffer exchange and concentration step - used for the preparation of the immunoaffinity column, also based on Sephadex (pre-packed, N-hydroxysuccinimide-(NHS)-activated, 1 mL bed volume). The immunoaffinity column was eventually blocked by 0.5 M ethanolamine and repeatedly used for the immunoaffinity ICP-MS experiments.

For the liquid handling, syringe pumps were used, connected by usual PEEK/Teflon tubing. The sample, the buffers, and pure water were filled in syringes and used subsequently. An important point is the extensive cleaning of the immunoaffinity column before use. For this purpose, 20 mL of phosphate-buffered saline (PBS, pH 7.4), 20 mL of PBS-Tween 20, and 10 mL of ultrapure MilliQ water were used.

For the immunological analysis of the eluted affinity fractions, an enzyme-linked immunoassay (ELISA) was employed, based on detection of ceruloplasmin by sandwich immunoassay. This immunoassay type is using two different antibodies for one analyte, leading to excellent structural selectivity and high sensitivity.

The quantification of protein immunoassays depends additionally on the availability of pure protein standards and hence is compromised, if such standards, not to speak about certified reference materials (CRM), are not available, which is the case for Cp.

An elegant way to circumvent this issue is the compound-independent calibration, which is often used in ICP-MS. Here, the calibration is performed with inorganic metal salts, irrespective of the binding form of the metal in the sample. In this work, we combined the strengths of two very powerful techniques, the extreme selectivity of affinity extraction with the excellent quantitation capabilities of ICP-MS, offering compound-independent calibration, sensitivity, and a very large dynamic range in addition to real-time signal compared to the post-run procedure in ELISA. This combination gives rich information about the performance of affinity enrichment of metalloproteins by comparison of the elution profiles and their comparative and quantitative examination. It has to be stressed that the offline-ELISA used in this work was performed only for the elucidation of the fate of the protein part of Cp. After the optimization of the experimental setup, ELISA would not be necessary for Cp determination anymore.

Sulfur is an abundant hetero-element of proteins, contained in the amino acids cysteine and methionine, hence this element was chosen as a non-specific protein signal. Cobalt was used as internal standard to normalize the ICP-MS signal. Gadolinium was added to the elution buffer to control the dead volume of the system. Thus, in our experiments, copper, sulfur, cobalt, and gadolinium were continuously monitored during the elution of Cp by ICP-MS.

ICP-MS analysis was performed on a sector field ICP-MS (Element 2, Thermo Fisher Scientific, Bremen, Germany). Mass traces of $^{63}Cu$, $^{59}Co$, $^{158}Gd$ and $^{32}S$ were recorded at medium resolution (R=4000), $^{59}Co$ and $^{158}Gd$ were used as internal standard and elution tracer, respectively. All standard elements are traceable to SRM from NIST (Merck, Darmstadt, Germany).

## 1. Preparation of chicken antibodies against human ceruloplasmin

Polyclonal chicken anti-human ceruloplasmin antibodies were produced at Davids Biotechnologie GmbH, Regensburg, Germany, by immunizing two chickens with native human ceruloplasmin (1 mg ceruloplasmin per animal). Ceruloplasmin from Human Plasma was obtained from Athens Research and Technology, USA, Prod. No. 16-16-030518). Eggs were collected for ten days and the egg yolk was extracted and subsequently affinity purified on immobilized ceruloplasmin. The antibodies were eluted with citrate/glycine buffer (pH 2.8) and neutralized immediately with phosphate buffer (pH 8). In total, about 25 mg of affinity purified antibodies were obtained.

## 2. ELISA protocol for antibody testing and offline ceruloplasmin determination

Microplates were coated with monoclonal anti-human ceruloplasmin antibodies (Pierce/Thermo, clone 3B11, antibody ID AB_1955530) diluted to 1:1000 in phosphate-buffered saline (PBS, pH 7.4, 100 µL per well) containing 10 mM sodium dihydrogen phosphate, 70 mM disodium hydrogen phosphate and 145 mM sodium chloride and incubated for 3 h at room temperature. The plates were sealed during all incubation steps with Parafilm. The plates were washed with buffer (pH 7.4) containing 45 mM potassium dihydrogen phosphate, 375 mM dipotassium hydrogen phosphate, 1.5 mM sorbic acid and 3% Tween 20 and then, the plates were blocked with 200µL per well PBS containing 0.1% casein for 30 min. After another washing step, 100 µL of a serial dilution of pure ceruloplasmin (product number 16-16-030518) purchased from Athens Research and Technologies was pipetted into the microplates and incubated for 1 h. As a sample, human serum, off the clot, Cat. No. S01049.1-01 from Merck was used. Following to washing steps chicken anti-human ceruloplasmin antibodies diluted to 1:5000 in PBS (100 µL per well) and incubated 1 h; 100 µL/well of enzyme conjugate (Santa Cruz Biotechnology, Heidelberg, sc-2428, goat anti-chicken IgY-HRP, 200 µg/0,5 ml) diluted 1:40.000 in PBS was added and incubated for 45 min. After washing, 100 µL/well of a freshly prepared substrate solution containing citrate buffer (220 mM potassium dihydrogen citrate, 0,5 mM sorbic acid sodium salt), hydrogen peroxide and tetramethylbenzidine was added. Color development was stopped after 10 min by the addition of 100 µL/well of stop solution (sulfuric acid) and read the absorbance at 450 nm.

## 3. Preparation of the immunocapture affinity column

An NHS-activated HiTrap HP (High-Performance Sepharose) column (1 mL, GE Healthcare Life Sciences) was used for the immobilization of affinity-purified chicken anti-human ceruloplasmin antibodies. First, the top cap of the column was removed and a drop of ice-cold 1 mM HCl was applied to the top of the column to avoid air bubbles. The syringe connector was attached and the snap-off end at the column outlet was removed. Isopropanol (in which the column was supplied) was washed out using ice-cold 1 mM HCl, (3 × 2 mL) at a flow rate of 1 mL min$^{-1}$. Approximately 10 mg of the affinity-purified antibodies were buffer exchanged and concentrated in 500 µL coupling buffer containing 0.2 M $NaHCO_3$, 0.5 M NaCl, pH 8.3 and injected onto the column. The column was sealed and incubated for 30 minutes at 25°C. Subsequently, residual NHS groups on the column were deactivated by 3 x 2 mL of buffer A containing 0.5 M ethanolamine, 0.5 M NaCl, pH 8.3 and of buffer B containing 0.1 M sodium acetate, 0.5 M NaCl, pH 4. Finally, the column was washed with 10

mL PBS and stored at 4°C until use. The chicken anti-human ceruloplasmin column was tested as follows. In a first affinity experiment pure ceruloplasmin (20 µg) was solubilized in 500 µL PBS and loaded stepwise into the column in 30 min. at RT. After loading (incubation) the column was washed with 5 ml of PBS followed by elution with 5 ml of 100 mM glycine pH 2.2. The elution fractions were collected in an ELISA microtiter plate (storage plate) drop by drop (about 35 µL). Prior collecting the elution fractions, the ELISA plate was filled with 200 µL PBS at pH 7.6 to avoid protein denaturation caused by the low pH of the elution buffer. In the elution step, the column was reversed to reduce rebinding of the protein in the lower part of the column and also to get a better elution peak shape. After the collection of the elution fractions the pH of the solution in ELISA plate was found to be about 7.1. Finally, the column was washed with 5 mL PBS and stored at 4°C for further use. The presence of ceruloplasmin in the elution fractions was detected by a direct ELISA assay (Fig. 1S).

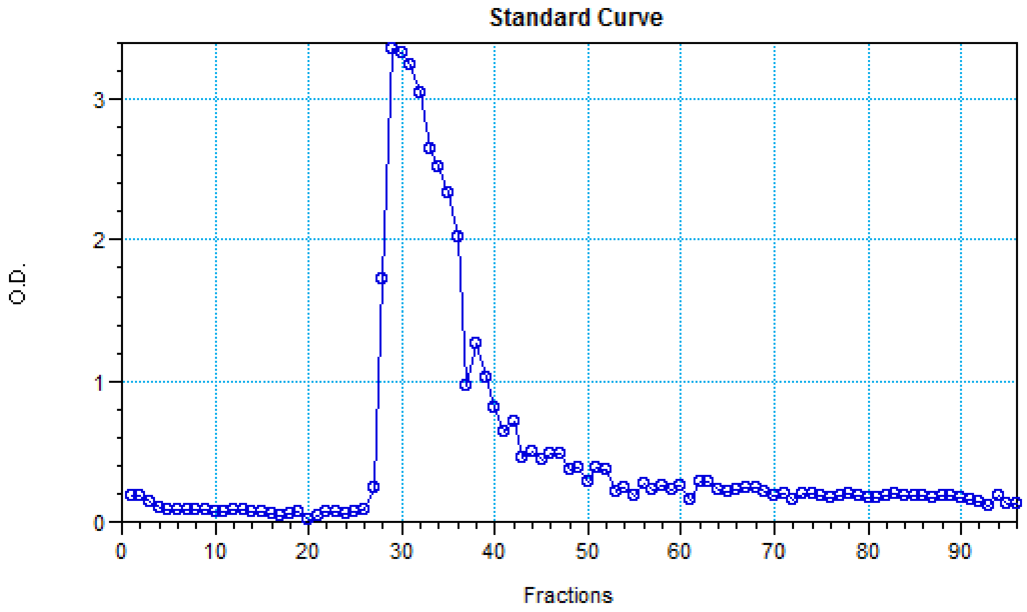


Fig. 1S: This profile shows that ceruloplasmin was eluted from the column in a sufficiently sharp peak.

## 4. ICP-MS protocol

ICP-MS analysis was performed on an Element 2 sector field ICP-MS (Thermo Fisher Scientific, Bremen, Germany). The conditions are shown in Table S1 (Additional File 1a). Mass traces of $^{63}$Cu, $^{65}$Cu and $^{32}$S were recorded at medium resolution (R=4000) and $^{59}$Co and $^{158}$Gd were used as internal standard and elution tracer, respectively. The isotopic ratio of $^{63}$Cu:$^{65}$Cu was found consistent through the analysis.

## 5. Protocol of the immunocapture ICP-MS experiments

The affinity column generated by immobilization of the chicken anti-human ceruloplasmin antibody on a solid support was connected to a splitter that divided the flow into two approximately equal parts; one used for collecting the fractions on an ELISA microtiter plate pre-filled with 200 µL of PBS (pH 7.6) to avoid denaturation of the eluted protein by the acidic conditions of the elution buffer. Every drop (about 35 µL) from the elution stream was collected in a separate well of the microtiter plate. The other flow went into the ICP-MS. Later the protein was detected by ELISA (Protocol see Section 2.). 100 µL of human serum diluted in 10 mL of PBS (pH 7.4) was loaded on the column at a flow rate of 30 µL $min^{-1}$. After loading, the column was washed using 50 mL of PBS followed by 30 mL of MilliQ water to avoid contaminating the ICP-MS with salts and to improve the baseline. The column was connected to two syringe pumps; one containing MilliQ and the other containing elution buffer (10 mM Glycine/HCl pH 2.2) both connected through a T-piece (Figure 1 of the main text). The flow rate was 1 mL $min^{-1}$. This construction enables changing the washing buffer to elution buffer without interrupting the flow while ICP-MS data is recording. The capillary that went to the ICP-MS was connected to another syringe pump by a T-piece introducing a cobalt salt as post-column internal standard (1 ppm cobalt in 2% $HNO_3$) at 25 µL $min^{-1}$.
